# Employments for Women

**Published:** 1892-07-16

**Authors:** 


### Employments for Women.

### IV.—LECTURING FOR THE COUNTY COUNCILS.

In the good old days—or shall we rather say, the bad old days?—you might be pretty sure, if you heard of an employment for women—for women of the upper classes, at any rate—that it was an employment which men considered to be quite beneath them—one which required no physical strength, little brain-power, and an inexhaustible faculty for sitting still, for, if it was work that involved running about, and seeing a little of the world, why, then it was not at all fit for women, whose proper place was their home. Now, however, things are quite different; and a very good thing it is that they are; for in the present day, when girls share the education once confined to their more fortunate brothers, to set them all down to everlasting darning or tapestry work, or even to a course of empty-headed, frivolous amusement, would probably lead to strange and alarming results. "How in the world did the girls of those old days manage to exist?" a girl of the present day has been heard to exclaim. "Why, if I had lived in those times, and had seen myself condemned to tapestry work in the old castle, while the men were out fighting, and having a good old time, I should either have gone out as an Amazon, or else drowned myself in the moat?" Happily, in these days, girls of an enterprising and impatient spirit are not reduced to any such alternatives. One by one, careers which were once considered only fit for men have been opened to women also; and within the last year or so, as we all know, yet another has offered itself, thanks to the County Councils, viz., that of giving simple lectures on cookery, nursing, laws of health, &c., all over the country, as these lectures may be required.

It would be impossible, we think, to find an employment more suitable to educated women than this, and it will specially appeal to lady-readers of this journal, most of whom, it may be presumed, know something of nursing; for one of the requirements of the National Health Society (to whom the County Councils constantly apply for lecturers), is that candidates for their lectureships shall have been trained for at least three months in a hospital or infirmary. There must be many among our readers who have had this amount of training, and yet have not taken up nursing as a regular profession. Perhaps they have only wished to qualify themselves for the better performance of home duties; perhaps they have found the constant strain of hospital work more than their delicate health would stand, and so have reluctantly been compelled to give it up. But in either case, they might very well feel that, the suitable training for it once obtained, lecturing for the National Health Society during a certain portion of the year was quite within their power; and we can hardly imagine a more useful or a more interesting employment than going round from village to village, and instructing the poor ignorant cottage-women in a series of "Homely Talks," how to make the cottage clean, healthful, and attractive.

Even to those who know nothing of hospital work, the course of training is not very formidable. The National Health Society (Berners Street) gives a course of instruction for teachers in sanitation, hygiene, elementary anatomy, physiology, home nursing, and first aid, the fee for which course, including examination, is £10 10s. Then a course of practical lessons on artisan cookery must be taken, the Society mentioning several schools which it specially "approves"; and finally there is the hospital or infirmary training already mentioned. This, however, says Dr. Schofield, one of the Society's regular lecturers, can in many cases be carried on at the same time as the attendance at lectures, so that the whole training can be easily completed in six months. This done, and the Society's examinations satisfactorily passed, suitable candidates will be selected in rotation for country lectureships, and we are told that their salaries will range from three to five guineas a week. We do not think, however, that a lecturer must expect to earn more than from eighty to a hundred pounds per annum, for, be it remembered, these classes can only be given as a general rule during the winter half of the year. This would be no drawback, but rather the reverse, to women who do not feel able to give up the whole of their time to their profession; while those who are under the necessity of earning all they can for themselves would at least have the summer months free for any other work they might find.

The course of instruction given by the National Health Society is also confined to the six months beginning with October and ending with March; so that it is impossible now for any intending student to get her diploma before March, 1893. But it is always well to take time by the forelock, and we can hardly doubt that some of our readers will be anxious to avail themselves of the opportunity for useful work thus offered.

What proportion the number of lecturers now bears to the number of lectureships, we cannot say; but that the next two or three years will see an enlargement of the work, we can hardly doubt. The country seems to be just waking up to a sense of its ignorance of these important matters—matters in which, as Dr. Schofield has told us, the girls in the quiet, unobtrusive little country of Belgium are instructed as a matter of course. We are beginning to see that with all our zeal—our very proper zeal—for education, we have not been going quite the right way to work; that the system of teaching practised until lately in our schools has resulted in making the boys—or, at any rate, the cleverer among them—think everything of book-learning, and nothing of intelligent application to manual labour—in making the girls look upon household management, cooking, and other domestic tasks, as only fit for their poor uneducated mothers, and quite beneath their own superior selves. But when once we English people have been stirred up, and made to see the existence of an evil, we will not be satisfied until we have done what we can to remedy it. Nothing can be better than that manifestly educated and refined women should take up these subjects, showing that they by no means disdain them, and proving that there is just as much scope for intelligence and thought in the management of a home as there is in the working out of a problem in mathematics, or in the reading of a new piece of music.

(*To be continued.*)

